# Giant Distal Ureteric Calculus in a Tertiary Care Center: Our Experience

**DOI:** 10.7759/cureus.104059

**Published:** 2026-02-22

**Authors:** Vinay S Kundargi, Siddanagouda B Patil, Santosh Patil, Anupam Banerjee

**Affiliations:** 1 Department of Urology, Shri BM Patil Medical College, Vijayapura, IND; 2 Department of Urology, BLDE (Bijapur Lingayat District Education) Deemed to Be University, Vijayapura, IND

**Keywords:** adult patients, giant distal ureteric calculus, hydroureteronephrosis, large ureteral stones, open surgery, stone clearance, ureterolithotomy, urolithiasis

## Abstract

Objective: This study aimed to describe the clinical presentation, diagnostic evaluation, and management of giant distal ureteric calculi at a tertiary care center.

Methodology: This retrospective case series included seven adult patients with distal ureteric calculi measuring >5 cm who were treated at a tertiary care facility between December 2023 and August 2024. Patients presenting with sepsis or acute renal impairment were excluded. All patients underwent comprehensive clinical evaluation and radiological assessment using ultrasonography and contrast-enhanced computed tomography, followed by open ureterolithotomy. Postoperative outcomes, including stone clearance and procedure-related complications, were documented.

Results: The mean age of the patients was 45.1 ± 19.3 years (range: 12-69 years). Females comprised five patients (71.4%) of the study population. The mean stone size was 5.81 ± 0.43 cm. Fever was observed in six (85.7%) patients, and three (42.9%) had positive urine cultures. Hydroureteronephrosis was present in all patients (7/7, 100%). Complete stone clearance was achieved in all cases following open ureterolithotomy, with no residual fragments or significant postoperative complications.

Conclusion: Open ureterolithotomy was a safe and effective treatment option for giant distal ureteric calculi, providing complete stone removal with minimal perioperative morbidity, particularly when minimally invasive approaches are not feasible or have failed.

## Introduction

Ureteric stones are a common cause of urinary tract obstruction [1], and large calculi located in the distal ureter pose significant challenges to both stone removal and preservation of renal function [2]. Stones measuring more than 1 cm are unlikely to pass spontaneously and generally require surgical intervention [3-6]. Delayed or inadequate treatment can lead to serious complications, including hydronephrosis, cortical atrophy, and irreversible renal damage [7].

Management of large distal ureteric calculi typically involves ureteroscopy with laser lithotripsy, often supplemented by adjunctive procedures such as ureteric stenting or balloon dilatation, particularly when standard techniques are unsuccessful [1,5,8]. Giant distal ureteric calculi are rare and pose distinct surgical challenges due to their size and location, often necessitating an individualized, stepwise management approach [9]. Despite their clinical importance and potential for severe complications, data on the clinical presentation, management strategies, and outcomes of giant distal ureteric calculi remain limited, particularly in tertiary care settings. This paucity of evidence represents a significant gap in the urological literature.

The present study aims to describe our institutional experience with giant distal ureteric calculi by detailing patient demographics and clinical presentation, evaluating diagnostic modalities, analyzing management strategies and surgical outcomes, and documenting perioperative complications and postoperative recovery.

## Materials and methods

Study design and setting

This retrospective case series was conducted in the Department of Urology at Shri BM Patil Medical College and Hospital, Vijayapura, India, by reviewing the medical records of eligible patients over nine months, from December 2023 to August 2024. The study was conducted after obtaining approval from the Institutional Ethics Committee (IEC) of Shri BM Patil Medical College, Hospital and Research Centre (Approval No.: BLDE (DU)/IEC-SBMPMC/150/2023-24).

Study population

Patients presenting with distal ureteric calculi measuring >5 cm were considered for inclusion. Adult patients (≥12 years) who underwent surgical management for stones >5 cm during the study period were included. Patients with sepsis, uncontrolled urinary tract infection, or acute renal impairment were excluded. Additionally, patients managed conservatively or by minimally invasive procedures were not included. Given the rarity of giant distal ureteric calculi, all eligible patients encountered during the study period were enrolled, yielding seven cases.

Clinical and radiological evaluation

All patients underwent a detailed clinical assessment followed by radiological evaluation. Abdominal ultrasonography was performed in all cases. Contrast-enhanced computed tomography (CECT) of the abdomen was subsequently used to confirm the diagnosis, accurately determine stone size and location, and evaluate the degree of hydroureteronephrosis.

Surgical management

Following radiological confirmation, all patients were planned for open ureterolithotomy due to the large size of the distal ureteric calculi, which rendered minimally invasive techniques unsuitable.

All procedures were performed under general anesthesia with the patients positioned supine. After standard skin preparation and draping, a lower abdominal incision was made to access the distal ureter. Careful, layer-by-layer dissection was performed to expose the ureter while preserving adjacent structures.

**Figure 1:**
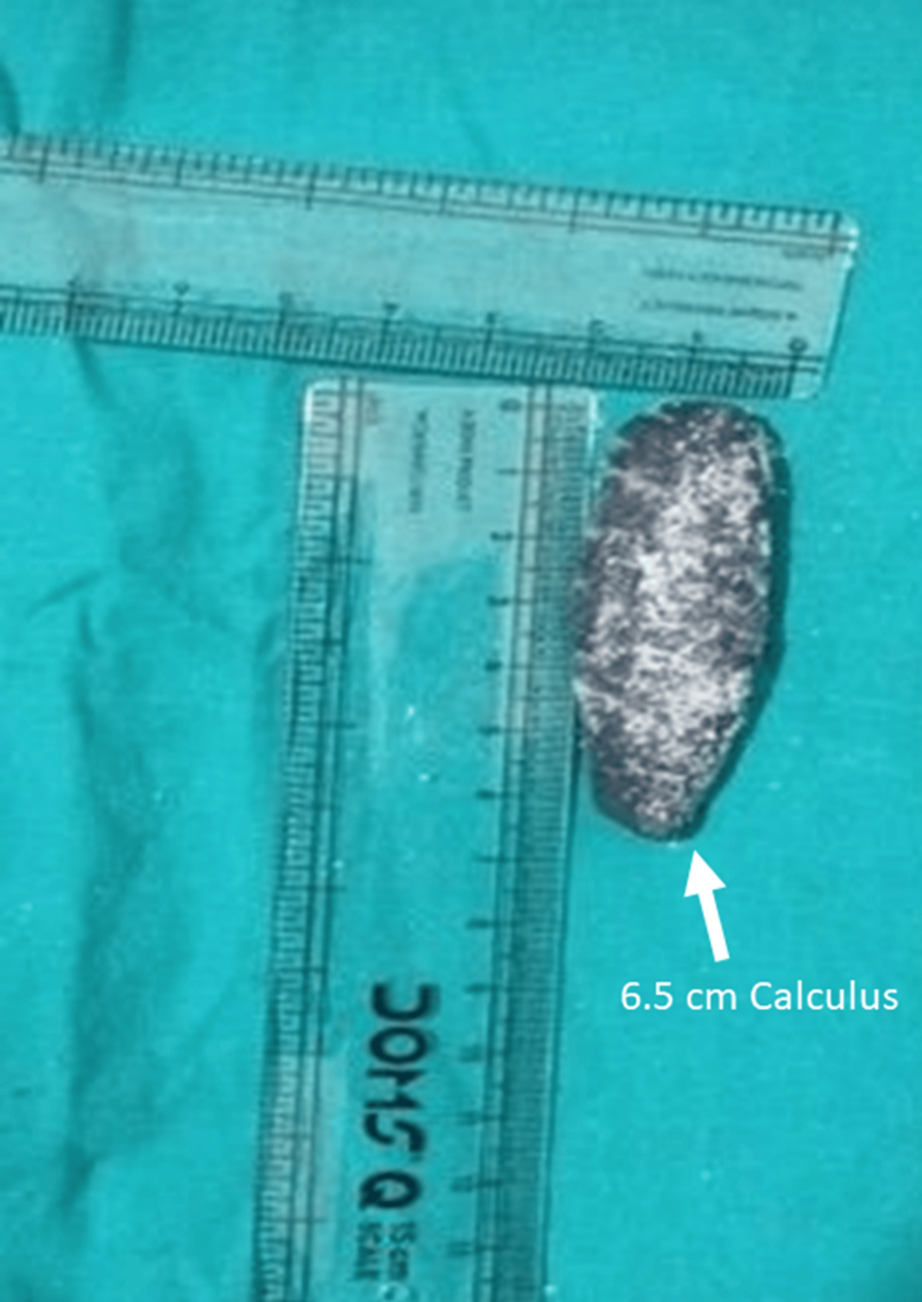
Intraoperative documentation and measurement of the extracted distal ureteric calculus

The ureter was identified and mobilized at the site of stone impaction, with marked proximal dilatation noted. A longitudinal ureterotomy was made directly over the palpable calculus. The giant ureteric stone was extracted intact using gentle manipulation and stone-holding forceps. The extracted calculi were irregular and elongated, measuring more than 5 cm in length, and their size was confirmed using a sterile measuring scale (Figure 1).

Following stone removal, the ureteric lumen was inspected for residual fragments and patency. A double-J (DJ) ureteric stent was placed in all cases to ensure adequate urinary drainage and facilitate ureteral healing. The ureterotomy was closed with absorbable sutures in an interrupted manner. Hemostasis was achieved, and the operative field was irrigated with saline. The wound was closed in layers, with drains placed where indicated. All extracted stones were photographically documented and sent for analysis when feasible.

Postoperative management and follow-up

Postoperative assessment included a kidney, ureter, and bladder (KUB) radiograph to identify residual calculi. DJ ureteric stents were removed after three to four weeks. Patients were monitored during the postoperative period to assess recovery, complications, and overall surgical outcomes.

Statistical analysis

Descriptive statistics were used to summarize the data. Continuous variables are presented as mean ± standard deviation (SD), while categorical variables are expressed as frequency (N) and percentage (%).

## Results

The mean age of the patients was 45.1 ± 19.3 years, with an age range of 12-69 years. Of the total seven patients, two (28.6%) were male, and five (71.4%) were female. The gender distribution did not demonstrate a statistically significant difference (p = 0.257) (Table 1).

**Table 1:** Demographic and baseline characteristics (n = 7)

Variables	Values
Age (years), Mean ± SD	12-69 (range), 45.1 ± 19.3
Gender	
Male (%)	2 (28.6%)
Female (%)	5 (71.4%)

The mean total leukocyte count was 12,186 ± 1,958/mm³. Renal function was preserved, with a mean serum creatinine level of 0.83 ± 0.18 mg/dL. The mean calculus size measured 5.81 ± 0.43 cm (Table 2).

**Table 2:** Laboratory and radiological parameters

Parameters	Mean ± SD
Total leukocyte count (/mm³)	12,186 ± 1,958
Serum creatinine (mg/dL)	0.83 ± 0.18
Calculus size (cm)	5.81 ± 0.43

Fever at presentation was observed in six (85.7%) patients. A positive urine culture for *Escherichia coli* was identified in three (42.9%) cases. Radiological evaluation revealed hydroureteronephrosis in all patients (7; 100%), while three (42.9%) patients demonstrated delayed contrast excretion. All patients underwent open ureterolithotomy (7; 100%), and no postoperative residual stone fragments were detected (0; 0%) (Table 3).

**Table 3:** Clinical, microbiological, and operative outcomes

Variables		N (%)
Clinical presentation	Fever at presentation	6 (85.7%)
Microbiology	Positive urine culture (*E. coli*)	3 (42.9%)
Radiological findings	Hydroureteronephrosis	7 (100%)
	Delayed contrast excretion	3 (42.9%)
Surgical management	Open ureterolithotomy	7 (100%)
	Residual postoperative stone	0 (0%)

**Figure 2:**
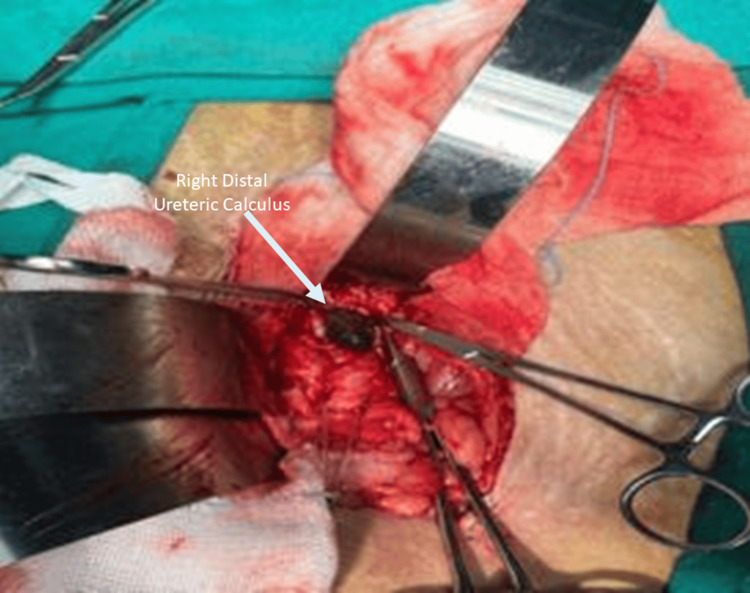
Intraoperative extraction of an impacted distal ureteric calculus during open ureterolithotomy

Figure 2 demonstrates adequate exposure of the distal ureter with successful intact stone extraction, highlighting the feasibility and effectiveness of open ureterolithotomy in the management of giant distal ureteric calculi.

**Figure 3:**
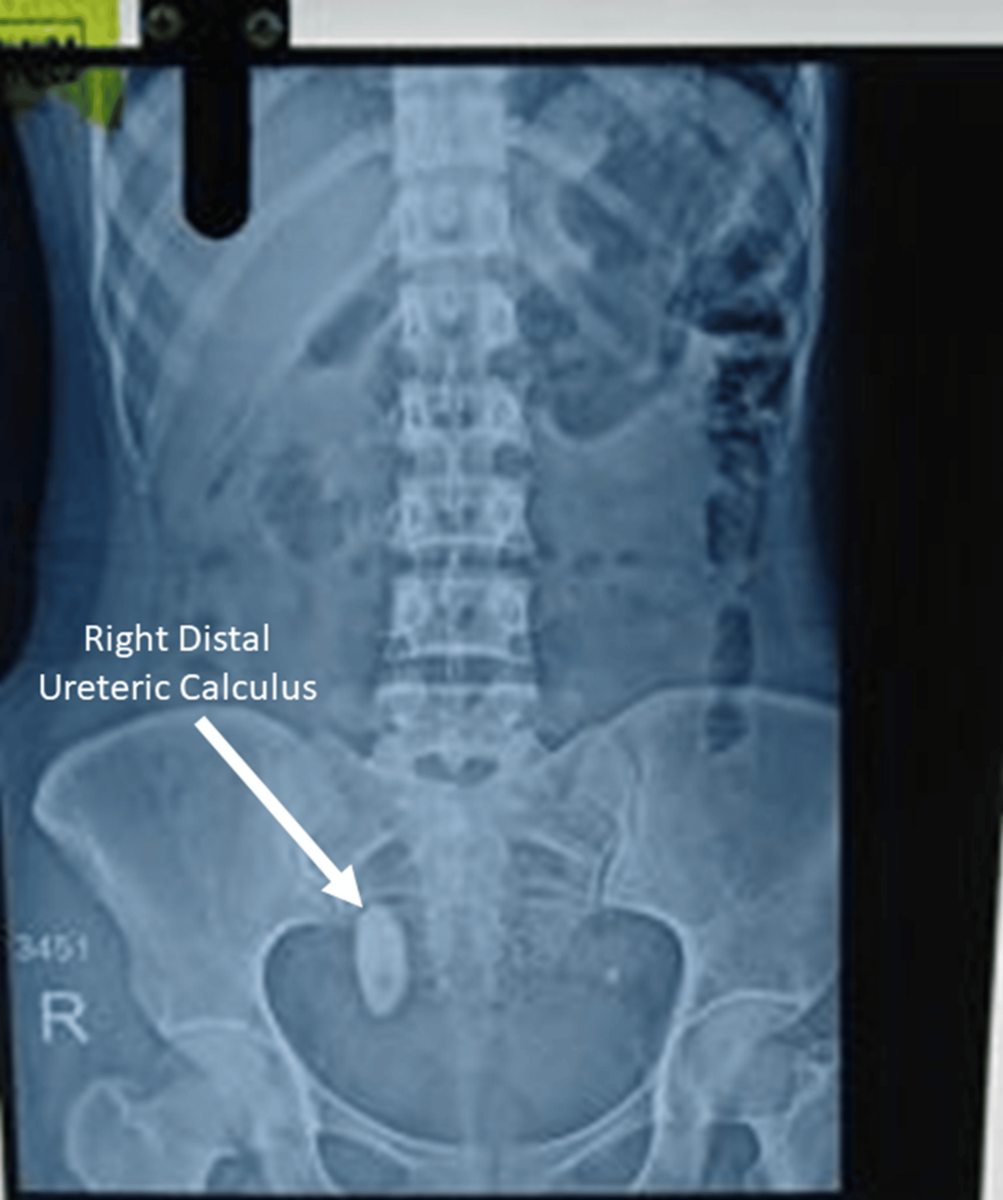
X-ray KUB showing right distal ureteric calculus KUB: Kidneys, ureters, and bladder.

Figure 3 represents an anteroposterior radiograph of the kidneys, ureters, and bladder (KUB) showing a large, well-defined radiopaque oval-to-elongated shadow in the pelvic region, located within the urinary bladder, slightly to the right of the midline, consistent with a vesical calculus. No additional radiopaque calculi are identified in the renal regions or along the ureters. The lumbar spine and pelvic bones appear unremarkable.

**Figure 4:**
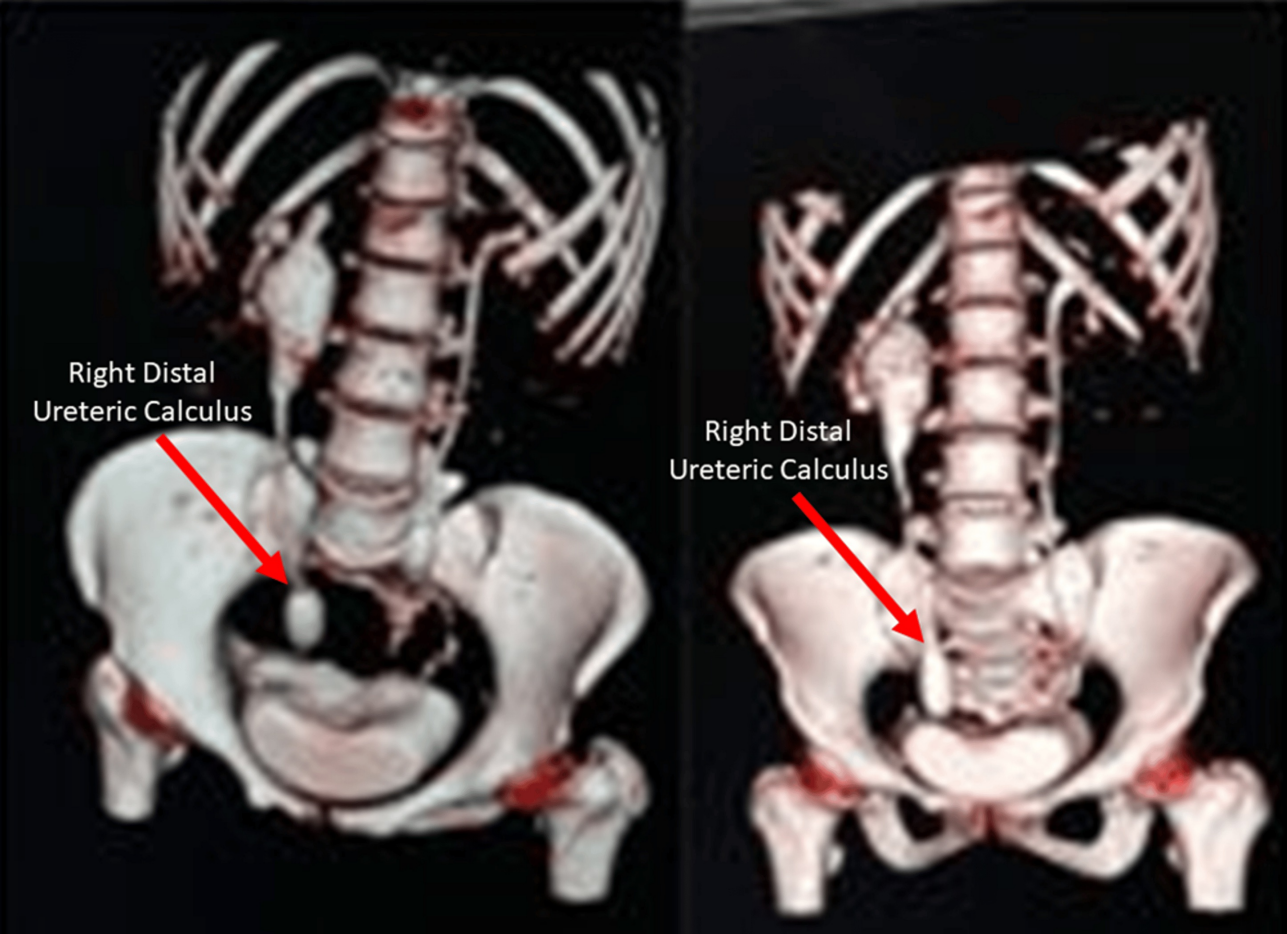
CT scan pelvis with 3D reconstruction image showing right distal ureteric calculus

Figure 4 presents three-dimensional volume-rendered CT images of the pelvis demonstrating a large, well-defined hyperdense calculus within the right distal ureter. The calculus appears elongated to oval in shape and is clearly visualized on multiple reconstructed views, confirming its distal ureteric location. The surrounding pelvic anatomy, including the lumbar vertebrae, sacrum, iliac bones, sacroiliac joints, and hip joints, appears intact, with no evidence of fractures, erosions, or destructive bony pathology.

**Figure 5:**
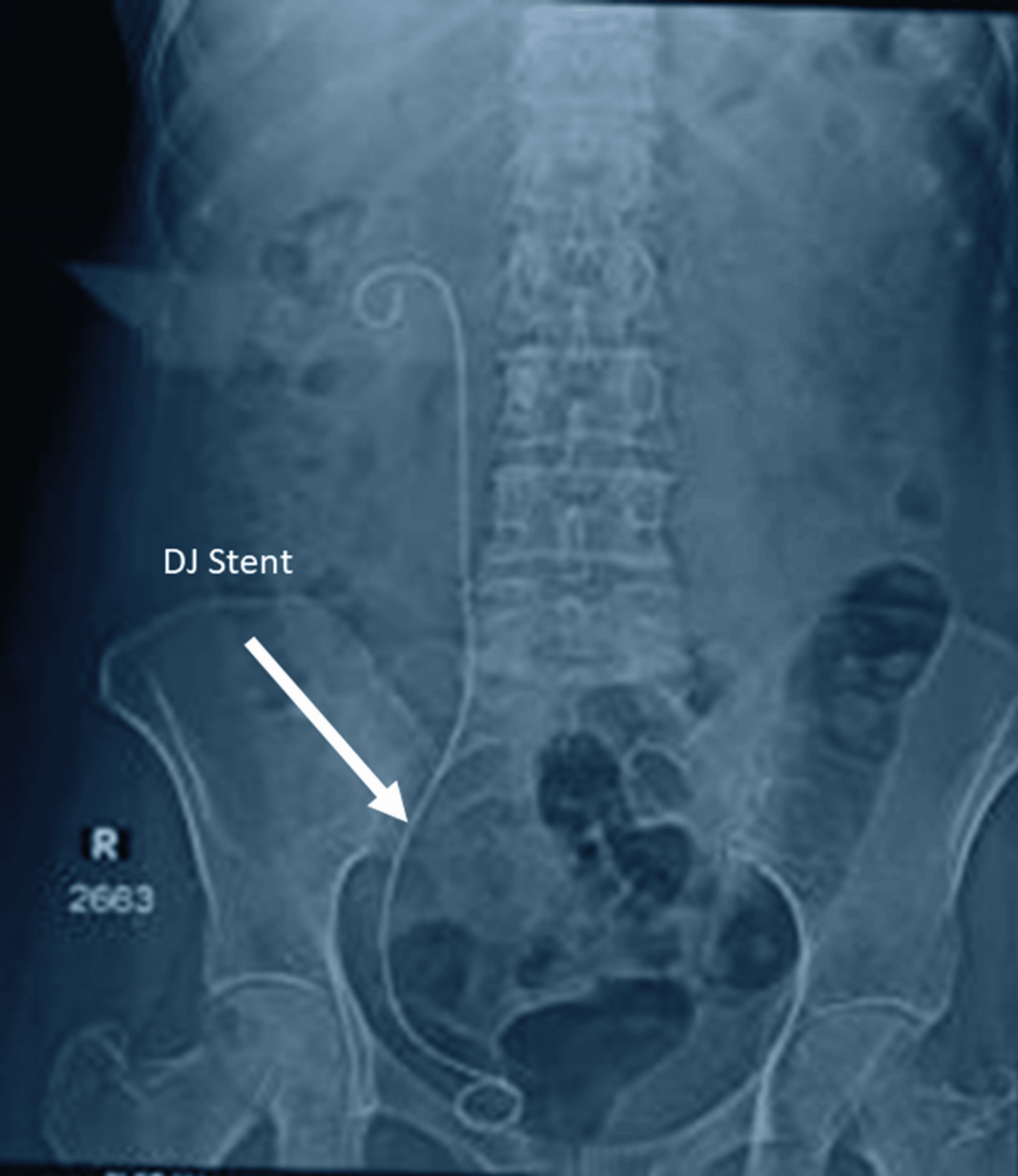
Postoperative X-ray KUB showing DJ stent in situ without any residual stone (stone clearance) KUB: Kidneys, ureters, and bladder; DJ: Double-J.

Figure 5 shows a postoperative KUB radiograph demonstrating a DJ ureteric stent in a satisfactory position, indicating adequate ureteric drainage, with no residual calculi, confirming successful surgical stone removal.

## Discussion

Giant distal ureteric calculi can occur across a wide age range, as demonstrated in our cohort (mean age: 45.1 ± 19.3 years; range: 12-69 years), affecting both sexes, with a female predominance (71.4% vs. 28.6% males; p = 0.257). Despite the substantial stone burden (mean size: 5.81 ± 0.43 cm), renal function remained preserved, with a mean serum creatinine level of 0.83 ± 0.18 mg/dL. However, systemic and infective manifestations were common, with fever present in 85.7% of patients and positive urine cultures for *E. coli* in 42.9%. Radiological evaluation revealed hydroureteronephrosis in all cases, and delayed contrast excretion was observed in three patients (42.9%). All patients underwent open ureterolithotomy, achieving complete stone clearance without residual fragments or significant perioperative complications, underscoring the safety and effectiveness of this approach.

Al Aswad et al. [10] reported a 21-month-old infant with a ureteric stone measuring 1.5 × 0.7 × 0.8 cm, successfully managed with laparoscopic ureterolithotomy, resulting in complete clearance without sequelae. Although there were substantial differences in patient demographics and stone size, both studies emphasize the importance of individualized surgical planning. While laparoscopy may be suitable for smaller or pediatric ureteric stones, our findings demonstrate that open ureterolithotomy remains a reliable option for managing giant distal ureteric calculi in adults, ensuring complete removal with low perioperative morbidity.

Recent guidelines emphasize that patient- and stone-specific factors are central to effective ureteric stone management, reinforcing the need for personalized treatment strategies [5]. Our findings support this approach, as surgical management was tailored to stone size, anatomical considerations, and clinical presentation. All patients underwent open ureterolithotomy for giant distal ureteric stones (mean size: 5.81 ± 0.43 cm), resulting in complete clearance. These results highlight the importance of a thorough preoperative assessment and the appropriate selection of the surgical modality, endoscopic, laparoscopic, or open, to optimize outcomes and minimize complications.

Jagannath et al. [11] reported that ureteric calculi predominantly affect individuals aged 31-50 years, with a male preponderance, and that the distal ureter is the most commonly involved site. They achieved success rates of 80%-100% using ureteroscopic laser lithotripsy (URSL) for distal stones and percutaneous nephrolithotomy (PCNL/PBPCNL) for proximal stones larger than 1 cm. In contrast, our study focused exclusively on adults with giant distal ureteric calculi (mean size: 5.81 ± 0.43 cm), predominantly females (71.4%), who were all managed with open ureterolithotomy. While minimally invasive techniques are effective for smaller stones, our findings suggest that open surgery remains a safe and dependable option for very large distal ureteric calculi.

A 2025 study [12] reported that ureteric stones most commonly occurred in males aged 30-50 years, with flank pain being the predominant symptom (87.1%). Extracorporeal shock wave lithotripsy (ESWL) was effective for proximal stones < 10 mm, and URSL achieved stone-free rates of 85.2% for stones up to 15 mm, albeit with higher postoperative complication rates (22.2%) in the URSL group. In contrast, our cohort primarily comprised adult females with giant distal ureteric stones, presenting predominantly with fever and hydroureteronephrosis, all of whom were successfully treated with open ureterolithotomy. This comparison reinforces that although minimally invasive techniques are appropriate for smaller stones, open surgery remains effective and safe for managing very large distal ureteric calculi.

The European Association of Urology (EAU) [3] recommends that the diagnosis of ureteric stones should incorporate clinical assessment, biochemical evaluation, and imaging, with ultrasonography as the initial modality and low-dose computed tomography as the gold standard. Treatment selection is guided by stone size, location, and composition and includes ESWL, ureteroscopy, and percutaneous nephrolithotomy, with medical expulsive therapy reserved for selected cases. Our findings suggest that for huge distal ureteric stones, conventional minimally invasive techniques may be insufficient, whereas open ureterolithotomy ensures safe and complete stone removal. These results align with guideline recommendations advocating individualized, stone-specific management strategies.

Sharma et al. [13] reported that giant ureteric calculi (>5 cm) are rare, with stones historically measuring up to 21.5 cm, and that open or laparoscopic ureterolithotomy has traditionally been the preferred treatment. Although minimally invasive approaches offer advantages such as reduced postoperative pain and faster recovery, our study demonstrates that open ureterolithotomy remains a practical option for adults with giant distal ureteric calculi, achieving complete clearance. These findings support Sharma’s [13] recommendation for individualized management based on stone size, location, and patient-specific factors.

A 2022 case report [14] described a 58-year-old patient with a large proximal ureteric stone (11 × 12 × 67 mm³) successfully managed with mini-endoscopic combined intrarenal surgery (ECIRS), achieving complete clearance. While their report focused on proximal stones treated endoscopically, our study addressed giant distal ureteric calculi in adults managed by open ureterolithotomy, with uniformly successful outcomes. Together, these findings emphasize that stone size, anatomical location, and patient characteristics are critical determinants of the optimal surgical approach.

Maranna et al. [15] highlighted that although endoscopic and laparoscopic techniques have largely supplanted open surgery for ureteric stones, open ureterolithotomy remains necessary in cases involving very large or impacted stones, anatomical abnormalities, or failed minimally invasive procedures. Consistent with these observations, our study demonstrates that open ureterolithotomy is a safe and effective option for managing giant distal ureteric calculi, achieving complete clearance without residual fragments or major complications.

This study has several limitations that should be considered when interpreting the findings. First, the retrospective study design limited control over data collection and may have introduced selection and information bias. Second, the small sample size (n = 7), reflecting the rarity of giant distal ureteric calculi, reduced the statistical power of the study and limited the generalizability of the results. Third, as this was a single-center experience conducted at a tertiary care institution, the findings may not be representative of outcomes in other clinical settings with different patient populations or levels of surgical expertise. Additionally, all patients were managed with open ureterolithotomy, and the absence of a comparison group undergoing minimally invasive procedures prevented direct comparison of surgical outcomes.

Long-term follow-up was limited, restricting assessment of late complications, recurrence rates, and long-term renal function. Furthermore, stone composition analysis was not available for all patients, which limited conclusions regarding metabolic factors and the risk of recurrence. We acknowledge that these aspects represent important areas for further research, and future studies with comprehensive follow-up and complete stone analysis would provide more robust evidence.

Despite these limitations, the study provides valuable insights into the management of a rare and challenging condition, supporting open ureterolithotomy as a safe and effective treatment option when minimally invasive techniques are not feasible.

## Conclusions

Giant distal ureteric calculi are rare but can cause significant compromise of renal function if left untreated. Our study demonstrates that open ureterolithotomy is a safe and effective treatment modality, achieving complete stone clearance with minimal perioperative complications. Careful preoperative evaluation and individualized surgical planning are crucial, particularly when stone size or anatomical considerations preclude minimally invasive approaches. Early diagnosis and timely surgical intervention are essential for preserving renal function and optimizing patient outcomes.
